# Exciting leak: Na^+^ background current makes chromaffin cells burst

**DOI:** 10.1113/JP281353

**Published:** 2021-02-10

**Authors:** Petronel Tuluc

**Affiliations:** ^1^ Department of Pharmacology University of Innsbruck Innsbruck Austria

**Keywords:** burst firing, chromaffin cells, NALCN, sodium channel

Chromaffin cells (CCs) of the adrenal medulla are neuroendocrine cells that secrete catecholamines (CAs) into the blood in response to stress. Canonically, it has been considered that CAs release is controlled though a cascade of events initiated by sympathetic nervous system. Upon splanchnic nerve activation, acetylcholine is released from the nerve terminals in the proximity of CCs, which activates the nicotinic acetylcholine receptors (nAChRs), leading to plasma membrane depolarization. The membrane depolarization activates the voltage‐gated Ca^2+^ channels, leading to increased global cytosolic Ca^2+^ concentration that promotes vesicle fusion with the plasma membrane and CA release. Nevertheless, accumulating evidence from several different laboratories has demonstrated that a subset of CCs display spontaneous activity and CA release in the absence of acetylcholine stimulation. These observations have been reported in both isolated CCs and tissue slices using intracellular or extracellular recording, thus excluding a possible experimental artefact. Biophysical characterization of CC plasma membrane conductances identified a large complement of voltage‐gated Na^+^, Ca^2+^ and K^+^ channels perfectly suited for the generation of spontaneous electrical activity with neuron‐like action potentials (APs) (Lingle *et al*. 2018). Interestingly, the CCs’ spontaneous electrical activity varies, with some cells displaying a tonic firing of individual APs while other cells fire bursts of APs. The burst firing mode has been demonstrated to be dynamically induced by splanchnic nerve acetylcholine release. Independently of the sympathetic nervous system activation, the burst firing can also be induced by acidosis, as lowering the extracellular pH results in membrane depolarization due to inhibition of pH‐sensitive TASK‐1/TASK‐3 and BK potassium channels (Guarina *et al*. 2017) or by ion channel expression levels (Vandael *et al*. 2010; Martinez‐Espinosa *et al*. 2014). Besides their classical roles in controlling cytosolic Ca^2+^ concentration and therefore vesicle exo‐ and endocytosis, high voltage‐gated Ca^2+^ channels have been proposed to occupy the central role in setting the CCs’ spontaneous and evoked electrical activity (Vandael *et al*. 2010).

A very elegant and thorough biophysical study coming from Nathalie Guerineau's laboratory, published in this issue of *The Journal of Physiology*, proposes a new molecular mechanism responsible for the dynamic switch between CCs’ tonic and burst firing modes (Milman *et al*. 2021). The authors characterize the CCs’ spontaneous electrical activity in acute slices of mouse adrenal glands and, in accordance with previously published reports, they identify that ∼60% of the cells exhibit a spontaneous activity while the remaining cells are electrically quiet. From the electrically active cells, half displayed a tonic AP firing pattern while the other half displayed burst firing. The AP derivative showed that the depolarization threshold and the resting membrane potential were significantly higher in bursting CCs, in agreement with previous data (Guarina *et al*. 2017). Importantly, Milman and colleagues also show that the same CCs can switch between continuous and burst firing mode depending on the resting membrane potential. Through a series of very well designed and intuitive biophysical experiments, the authors show that a Na^+^ depolarizing background conductance operating at resting membrane potential is involved in this behaviour. Using well established pharmacological tools (TTX, ouabain, caesium) the authors reveal that this background current is not conducted by very obvious candidates like voltage‐gated Na^+^ or K^+^ ion channels (Na_V_ K_V_) or by the Na^+^/K^+^‐ATPase pump, but instead it represents a hitherto unidentified CC Na^+^ conductance. *In situ* hybridization analysis showed that the Na^+^ leak channel NALCN is expressed in CCs, suggesting that its conductance is responsible for a more depolarized CC resting membrane potential.

The study is very timely and has high physiological relevance because the CCs’ electrical activity pattern dramatically affects the amount of CA release. Previously it has been shown that during burst firing mode the CA release is ∼3.5 times higher compared to the amount released during single APs (reviewed in Lingle *et al*. 2018). The results of Milman and colleagues further cement the notion that CCs of the adrenal medulla display a spontaneous electrical activity that is independent of sympathetic nervous system activation. The observation that the same cell can switch at ease between tonic and burst firing mode suggests that all CCs express a similar Na_V_, Ca_V_, K_V_ ion channel complement. The only difference is the Na^+^ background current (e.g. NALCN expression) which sets the resting membrane potential deciding the firing mode. It is tantalising to speculate that CCs of the adrenal medulla are not a uniform population, with some cells having a higher NALCN expression and therefore being more prone to burst firing or even working as pace‐maker cells that initiate the electrical activity that afterwards can spread through gap junctions to neighbouring cells. Single cell RNA‐seq has provided ample evidence of heterogeneous cell populations in many cell types (e.g. neurons, pancreatic β‐cells) and it is only a matter of time till the same questions will be asked in CCs.

This inspiring study shows once again that a well‐established neuroendocrine model system like CCs, which has delivered so much information on vesicle exocytosis and cell excitability during the past few decades, still holds many exciting secrets ready to be uncovered.

## Additional information

### Competing interests

The author declares no conflict of interest.

### Author contributions

Sole author.

### Funding

This work was not supported by any funding agency.
